# Integrating digital therapeutics for patients with mental illness into clinical practice: perspectives for US healthcare professionals

**DOI:** 10.3389/fpubh.2026.1817617

**Published:** 2026-09-02

**Authors:** Brandon M. Kitay, David C. Mohr, Stephen M. Schueller

**Affiliations:** 1 Department of Psychiatry and Behavioral Sciences, School of Medicine, Emory University, Atlanta, GA, United States; 2 Department of Preventive Medicine, Center for Behavioral Intervention Technologies, Northwestern University Feinberg School of Medicine, Chicago, IL, United States; 3 Department of Psychology, University of California Irvine, Irvine, CA, United States

**Keywords:** clinical practice, clinician perspectives, digital therapeutics, implementation science, mental health

## Abstract

As the landscape of digital health tools has expanded, digital therapeutics have emerged as promising treatment options for various mental health conditions. Although digital therapeutics have demonstrated efficacy in prevention, treatment, and management of mental illness, adoption of these interventions in clinical practice remains low. Here, key questions for healthcare providers about implementation of digital therapeutics into US clinical practice are addressed, focusing on digital therapeutics for mental illness. The landscape of digital therapeutics is described and current barriers to adoption across stakeholders are discussed, considering patients, providers, and health systems. This article further highlights opportunities for development and population-based implementation of digital therapeutics.

## 1 Introduction

The use of digital health tools to improve quality and access of healthcare in the United States is increasing, particularly as evidence supporting the effectiveness of digital therapeutics (DTx) grows (1, 2). DTx are defined as “health software intended to treat or alleviate a disease, disorder, condition, or injury by generating and delivering a medical intervention that has a demonstrable positive therapeutic impact on a patient’s health” (3). DTx employ available technologies, including smartphones, browser-based tools, messaging, or virtual reality to deliver evidence-based interventions as part of patient care (1). Categorized under “Software as a Medical Device” by the US Food and Drug Administration (FDA), DTx undergo regulatory review and oversight including authorization/clearance for an indication (prescription or over-the-counter) (2, 4–6). They are typically evaluated in randomized controlled trials to “verify the clinical safety, performance and effectiveness when used as intended by the manufacturer” (4). DTx differ from health and wellness applications (apps) that have no requirement for clinical evidence, are not subject to regulation, and are often marketed direct-to-consumer (Supplementary Figure 1) (1). DTx may require prescription/referral by a qualified healthcare provider (HCP) who oversees its use by patients (5).

DTx have demonstrated effectiveness including decreasing symptom burden, improving cognition, and supporting medication adherence or functional outcomes across various disorders and serious mental illnesses (e.g., major depressive disorder, post-traumatic stress disorder, panic disorder, and psychosis) (7–25). Several DTx have FDA marketing authorization, either *de novo* authorization (novel devices) or clearance (devices substantially equivalent to an authorized device and have undergone the 510(k) premarket notification process). FDA-authorized/cleared products are presented in Supplementary Table 1 (15–33). The Centers for Medicare and Medicaid Services (CMS) introduced Healthcare Common Procedure Coding System (HCPCS) codes, which may be used by any HCP to account for the distribution of an FDA-cleared Digital Mental Health Treatment (DMHT) device and for provider time to support longitudinal management (34, 35).

DTx for psychiatric disorders are often developed with input from mental health professionals and patients to enhance end-user experience and likelihood of adoption. Features of DTx may include self-directed training (e.g., psychoeducation and therapeutic skills practice), treatment reminders, behavior-modifying gamification or contingency management, goal setting, and progress tracking. Content of DTx may be general for all users or personalized based on patient-reported outcomes, preference, or task completion/performance (e.g., personalized treatment protocols).

DTx may empower patients to take an active role in their care by providing user-friendly, personalized treatments accessible at convenient times (36). With the prevalence of mobile device access across patient populations (37, 38), these interventions may help overcome workforce-related barriers to access, including lack of local or regional mental health specialists, restricted insurance-based services, and time constraints (e.g., family and work commitments) (39). DTx may support practitioner-guided psychotherapy between traditional office visits, improving efficiency and quality of care (40–42). Thus, DTx may provide a scaffold for treatment plans and anchor points for objective treatment goals.

When using DTx in practice, several issues must be considered, including liability, data use and protection, privacy, maladaptive use, and sustainability (43–45). Due to the recency of DTx, rapid development timeline, and novel FDA clearance pathway, long-term effects, durability of benefit, and potential harms must continue to be explored (44). Harms from use may not be limited to adverse events documented in traditional pharmacotherapy clinical trials. Potential risks associated with use of DTx include loss of privacy, inequity/discrimination, social/personal loss, or physical injury (46). Potential long-term risks include user-dependence, problematic use, unintended changes in behavior (47). Given that DTx constitute a novel therapeutic modality, approaches to evaluation, regulation, and deployment continue to evolve; a central challenge lies in determining the degree to which traditional models, originally designed for pharmacological or psychosocial treatments, can be appropriately adapted to address the distinctive features that differentiate DTx from conventional therapeutic approaches.

Despite increasing evidence supporting the efficacy of DTx for mental health conditions, there remains a critical gap in understanding how these interventions can be effectively implemented in routine clinical practice. Existing literature has focused largely on clinical effectiveness, with comparatively little attention to practical implementation strategies, workflow integration, reimbursement mechanisms, and stakeholder-specific barriers (7–25). This article addresses this gap by synthesizing current evidence, recent policy developments, and implementation experience to provide actionable recommendations for integrating DTx into U. S. mental health care settings.

This article aims to provide insights into adoption and implementation of DTx in US clinical practice, discussing features, benefits, and challenges from the patient and HCP perspective. These insights come from the authors’ experiences using and implementing DTx in various contexts and settings.

## 2 Approach to the review

This narrative review and clinician perspective synthesizes the current literature on DTx in mental health and discusses practical considerations for their integration into US clinical practice. The review was informed by published peer-reviewed literature, clinical guidelines, and regulatory documents identified through the authors’ knowledge of the field and targeted literature searches. As the objective was to provide a clinical perspective rather than a systematic evidence synthesis, a formal systematic review methodology was not employed.

## 3 Common questions for HCPs about DTx

Translating evidence into clinical practice is rarely straight-forward, particularly when it comes to emerging therapeutic modalities such as DTx. The following section takes a practical, question-driven approach to guide clinicians through the key considerations for integrating DTx into routine clinical practice for mental health care.

### 3.1 How should HCPs, administrators, and systems consider the role of DTx in delivery of mental health services?

In the US, it is estimated that about a quarter of adults have had any mental illness, with ~6% having experienced serious mental illness (48). Furthermore, 122 million people live in designated Health Professional Shortage Areas (HPSAs) for mental health, with an estimated shortage of 6,200 mental health practitioners (49). Of these HPSAs, 62% are in rural locations (49). Indeed, most rural areas in the US do not have psychiatrists (50). DTx have potential to improve access to mental health services and reduce healthcare disparities by overcoming geographic barriers, as well as offering more acceptable entry points into mental health treatment (51). People with mental illness may face stigma due to religious/cultural beliefs that prevents them seeking treatment (52–55). Patients may be more open to treatment via digital interventions as a discreet option over traditional referral care pathways (56, 57). Furthermore, DTx can increase accessibility of services to populations underserved due to communication barriers such as the deaf/hard of hearing and non-native language speakers who require translation services (58–60).

In addition to improving access for underserved populations, administrators and clinicians may consider DTx to optimize healthcare resource utilization and efficiency (61, 62). For example, DTx may provide opportunities for stepped care approaches by delivering interventions better matched to the severity of current symptoms and affording more time for clinicians to engage directly with patients requiring higher intensity care (63, 64).

### 3.2 What are some of the systemic challenges and potential solutions for adopting DTx in the clinic?

Systemic barriers to DTx adoption include absence of formal implementation and workforce training, technical challenges with incorporation into clinic- or electronic medical record (EMR)-driven workflows, cost-prohibitive startup and reimbursement models, and low financial incentivization to drive clinician adoption. Addressing these barriers requires an initial investment to support DTx integration, including addressing issues of coverage and payment, developing training programs and supporting continuing education, and working with clinicians to mitigate excessive additional workflow burden.

HCPs may benefit from continuing medical education courses on DTx to understand their novel mechanisms of action, role in multimodal treatment plans, and integration into modern clinical practice. Such courses are presently offered by professional organizations including the Digital Therapeutics Alliance (65), American Psychological Association (66), and American Psychiatric Association (67). The Society for Digital Mental Health’s DMHT Implementation Workgroup has developed an initial playbook describing practices that have been successful for implementing DMHTs in US healthcare settings (68). Clinicians may require time to review patient data from DTx to use in their day-to-day patient care and periodically become acquainted with new product information (e.g., expanded indications, software updates).

Clinicians, particularly those in primary care, may not have time during a typical visit to adequately onboard a patient to a DTx. Administrators should consider whether they can make “digital care navigators” (personnel providing technological support) available. This role may additionally involve promoting digital literacy, triaging patients to the most appropriate intervention, problem-solving around technology access, assisting patients in downloading and onboarding, promoting longitudinal engagement through a DTx treatment course, and liaising with product support (69–71). A major barrier to successful initiation of a DTx is that patients often do not register or download the product following a prescription or referral (72). Timely, proactive engagement by digital care navigators increases patient registration conversions, although effectiveness drops if assistance is provided 24 h post-referral (68, 73).

DTx may require a prescribing or referring HCP, as many of these tools supplement psychotherapy and/or medication management, especially if reimbursement will be sought via newly developed codes for DMHT Medicare coverage (35, 74). There are three codes: one covering supply of DMHT devices and providing patient education/onboarding, two covering monthly treatment management services including review of data generated by the device and communication with patients (35). These codes provide structure for institutions to begin implementing DTx. Commercial payors often follow CMS coverage decisions and use similar coding frameworks; however, commercial payors have not widely adopted new codes (75).

### 3.3 How should a DTx product be selected?

Evaluating DTx includes several important considerations. Comprehensive evaluations include assessing appropriateness for the therapeutic target, the evidence-base supporting efficacy or effectiveness in real-world application, access, privacy, safety, usability, and provision of data (68, 76).

Providers should assess whether the app comes from a reputable source, including evaluating company viability, ownership, and funding sources. Regarding access, providers must consider hardware requirements (e.g., smartphone, tablet, computer), compatibility with the patient’s personal devices and accessibility features, whether peripheral hardware is required (e.g., wearable devices, sensors), and if the DTx function is internet dependent (e.g., may be accessed or work offline). Data protection is another key consideration; if the DTx collects, uses, or transmits sensitive data, this should be done securely, and include a transparent privacy policy.

Providers should also evaluate the intended use and mechanism of action of a product to match the right patient to the right tool at the right time. DTx can be designed to produce mental, behavioral, or physiological changes, provide support with disease management, teach therapeutic skills, provide psychoeducation, and more. Mechanisms to achieve these outcomes include behavioral therapy, biofeedback, cognitive training, software-directed disease management, and software-led clinical coaching (76, 77). Thus, DTx may be designed for a specific diagnosis and validated in a specific patient population or may offer interventions for common symptoms across psychiatric diagnoses (e.g., insomnia or sleep quality). Providers may also consider the value DTx can provide to caregivers and their social support network (76, 78).

When selecting a DTx, providers should review both clinical and real-world data to assess safety and effectiveness, as participants in a research study may be more motivated and engaged with a DTx than typical patients in the real world. Additionally, providers should be aware that the clinical evidence supporting DTx varies widely in quality, with some trials limited by poor study design, small sample sizes, weak or poorly defined control conditions, and a lack of long-term follow-up. Providers should interpret effectiveness claims cautiously until more rigorous, longer-term research is available. Evidentiary standards related to DTx are still evolving and future work must consider how best to incorporate digital endpoints, post-marketing surveillance data, and other evidence, that encompass both the intended therapeutic benefits and unintended consequences, to inform a comprehensive and rigorous benefit–risk assessment. Ease of use is essential for patients and providers to realize the benefits of a DTx. For patients, the app’s interface and overall functionality drive initial adoption and long-term engagement. For providers, it is important that data are easily shared and presented for patient- or population-level interpretation. Data sharing with EMRs is a useful feature, though such integration is uncommon.

Resources to support DTx selection are available. These include the American Psychiatric Association App Advisor and the Quality Framework to Assist Stakeholders in Technology Evaluation for Recovery to Mental Health and Wellness, which provide detailed models for evaluation of DTx apps (76, 78). Recently the American Psychological Association launched the Digital Badge Solutions Library, a searchable resource to guide clinicians, health systems and the public to evidence-based digital mental health tools. The digital badge is awarded to technologies that submit for evaluation and meet proprietary criteria addressing regulation and safety, data protection and privacy, usability, and accessibility (79). The M-Health Index and Navigation Database provides a searchable library of many digital health apps; each entry includes information based on the American Psychiatric Association App Evaluation model, giving a view of app characteristics which supports informed decision-making (80).

Adoption of DTx by health systems/payors depends on the economic benefit and/or demonstrable return on investment (61). Considerations for economic evaluation include costing models and economies of scale. DTx may be offered with a unit price-based pricing model, based on the cost of the product and volume of use, or value-based contracting in which the overall price is based on outcomes or savings (61, 75). Given DTx are new technologies with emerging evidence regarding real-world effectiveness, safety, and impact on healthcare cost offset or savings, their scope of coverage and reimbursement remain unknown variables (61, 81). A modified Delphi study found payors were most likely to review a DTx product if it was FDA-reviewed, required a prescription, would be paid for using premium dollars, treats rather than diagnoses or monitors a condition, had a low clinical opportunity cost, or could address population health metrics (82). From January 2025, HCPs are able to bill Medicare directly for DMHTs; specifically, DMHTs may be prescribed/ordered by HCPs authorized to diagnose and treat mental illness (35, 74).

### 3.4 Considering the spectrum of mental illnesses, is there any consensus on considering DTx for a patient with a mental illness?

No current consensus exists on if/when to consider initiating a DTx for a mental illness. Broadly, the decision to initiate a DTx will require detailed understanding of the indication, context under which the DTx was studied, and intended benefits or use, like the decision-making for other evidence-based treatments. DTx with limited provider involvement should be reserved for mild-to-moderate, low-risk cases, whereas higher-risk or severe cases require DTx to supplement human-delivered care, with regular follow-up. Most current digital interventions are not intended to support patients with active suicidal ideation; however, there are technologies in development emerging as useful suicide prevention tools, with approaches including psychotherapies, psychoeducation, creation of safety plans, real-time monitoring of symptom severity, and connection to emergency resources (83–89). Some DTx have specific indications in relation to concomitant therapy, such as Rejoyn, indicated for patients with major depressive disorder receiving antidepressants (90, 91). For some DTx, it may be ideal to initiate a patient when they are receiving inpatient care, for example, to mitigate risk of relapse or recurrence through supporting care transitions. When deciding whether to initiate a DTx for a patient, providers should consider the patient’s willingness to engage with DTx, their personal perception and attitude towards digital interventions, and digital literacy levels (92, 93). It is important to acknowledge that a DTx may not meet the needs of some patient populations, for example, due to a lack of validation in that patient population or culturally informed development (93). Ultimately, shared decision-making is important when considering initiating a DTx.

### 3.5 How should HCPs describe DTx to patients?

**Figure 1:**
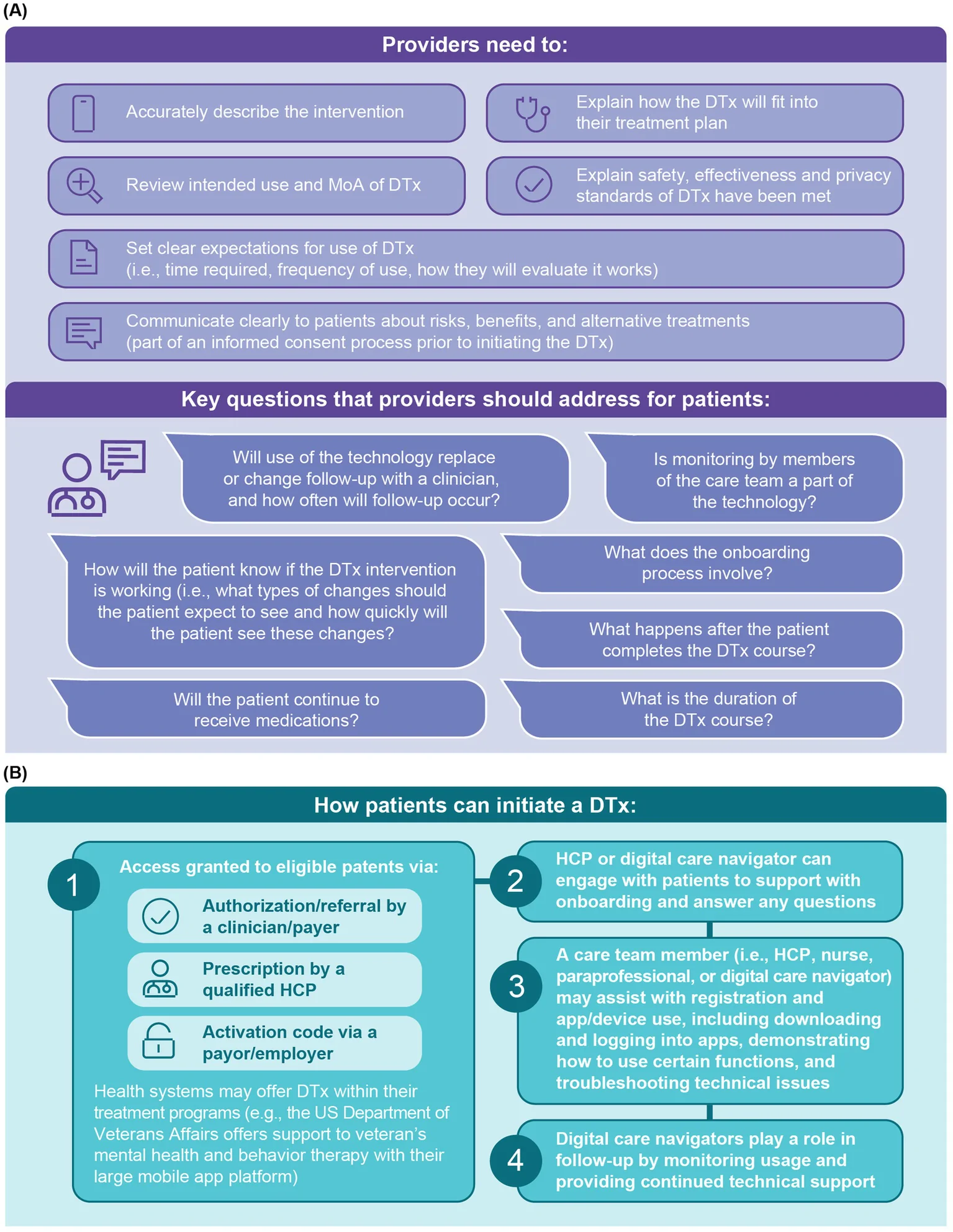
How HCPs should describe DTx to patients **(A)** and how patients can initiate a DTx **(B)**.

Patients may have limited knowledge or pre-existing biases related to the role of technology in medical care or digital mental health tools. As such, providers must provide clear explanations to their patients to address prior perceptions or lack of knowledge (68) (Figure 1A).

### 3.6 How does a patient initiate a DTx as part of their care?

The process for initiating a DTx as part of a patient’s care is presented in Figure 1B.

### 3.7 What are some of the patient concerns with initiating a DTx?

Patients may have concerns about the effectiveness of DTx relative to standard-of-care; they may perceive DTx as an inferior treatment option compared with in-person psychotherapy and have difficulty seeing how a DTx might help them (92–94). Patients also tend to be worried about the logistics of a DTx, for example, what it involves, how to navigate onboarding, what daily time commitment is required, what technical proficiency is needed, how to manage technical issues (i.e., patients often ask who they should contact for help) (92, 93). Patients may also have questions about integrating the DTx into their daily routine, including what to do if they are having difficulty engaging with it. Similarly, patients may have concerns about how the DTx will be integrated into the treatment plan, including the plan for follow-up with a clinician (36) and what to do if their symptoms do not improve within the timeframe of anticipated benefit. Another important issue for patients is cost, primarily if the DTx is not a covered benefit or coverage is unknown (94). Privacy of health data may also be of concern, particularly regarding what data are collected (especially from those with mental illness), who has access to the data, how data are used, and what data protection measures exist (36, 94, 95). As DTx integration into clinical practice expands, data privacy and security become of paramount importance requiring compliance with Health Insurance Portability and Accountability Act (HIPAA), user consent, data encryption and protection, and adherence to Federal Trade Commission mandates governing risk assessment, incident response, and breach notification protocols (68). In lieu of standardized cybersecurity regulations for DTx to minimize data breaches, providers should be mindful that few DTx devices have built-in cybersecurity protections leaving them potentially vulnerable to such breaches (96). It is critical to anticipate and be prepared to address these concerns before initiating the onboarding process.

### 3.8 What can providers do to maintain patient engagement with DTx?

**Figure 2:**
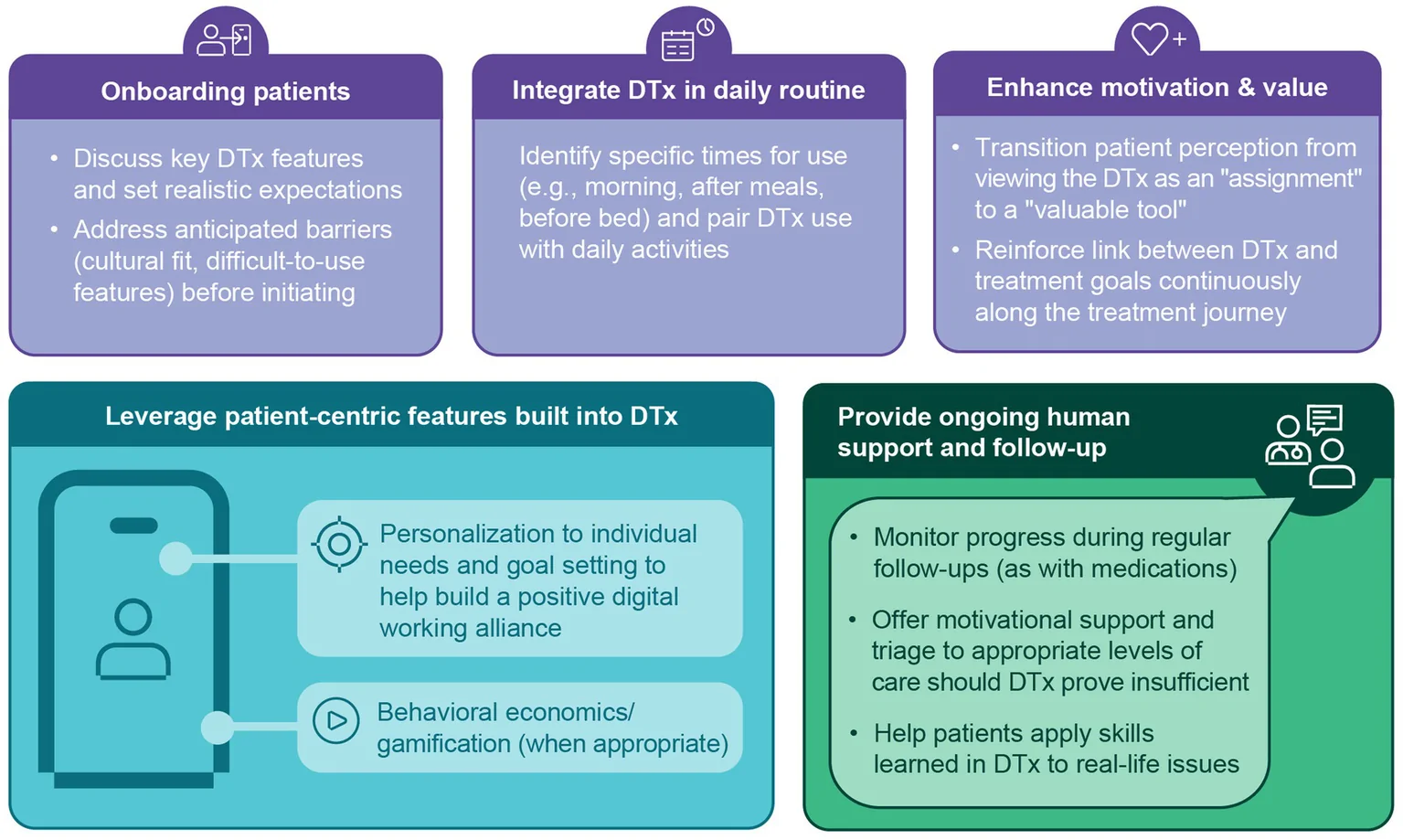
Key strategies to support patient engagement with DTx.

Sustained patient engagement can be challenging to achieve for DTx courses of longer duration, and the benefits of the DTx may not be realized without consistent use. Figure 2 outlines key strategies providers can use to support ongoing patient engagement with DTx.

### 3.9 What are some of the considerations for monitoring patients?

DTx can involve different levels of support from providers, and this support may be delivered by different experts, including HCPs or digital care navigators. Support of DTx may be for adherence only, as outlined in the supportive accountability model, which is limited to setting patient expectations and monitoring engagement to promote adherence and improve engagement outcomes (97). Further support may be provided to patients using the efficiency model to ensure they understand the concepts of the DTx, that DTx tools are a good fit, and that they are integrating it into their daily lives (98). A DTx can be integrated into traditional treatments with a blended care model, where both clinicians and a DTx are working in conjunction (99). Aspects of blended care design, including frequency of follow-up and which member of the care team follows up (e.g., MD, PhD, PsyD, licensed clinical social worker, digital navigator/coach), may vary across DTx and healthcare delivery settings.

Other considerations include passive versus active monitoring features of DTx and digital- versus human-led monitoring. Passive monitoring (e.g., wearables) provides objective data to complement patient-reported outcomes (e.g., survey completion or mood screening) or provide insight into disengaged patients, but it also raises privacy concerns and can be challenging to manage/interpret. Digitally-led care (e.g., text message reminders) enables automation and scalability, allowing more frequent and convenient patient communication. Human-led care enables more personalization and is more effective at promoting engagement but cannot be deployed as frequently or easily. In a blended care approach, the strengths of both approaches can be leveraged to provide maximal benefits: patients receive between-visit support from DTx while providers handle informed consent, engagement, safety monitoring, and referral to higher levels of care when needed.

### 3.10 How can providers best advocate for patients to have access to DTx?

Providers can promote access to DTx in a variety of ways (43) and may need to advocate for the time, training, and infrastructure resources needed to effectively deliver and support DTx treatment (100). Providers may participate in clinical trials to advance development of DTx, and work to establish best practices and guidelines for integration and use of DTx in clinical practice. While CMS has established HCPCS codes to support documentation and reimbursement, a major barrier to DTx consideration involves unanswered questions about coverage, reimbursement, and cost; engaging payors and policymakers will be critical for progressive adoption (81). The Aimed Alliance has the ACTION initiative for DTx that is working to expand DTx adoption, coverage, and access for people with chronic diseases, and has a working group focused on mental health (101). In the US, awareness of and coverage for DTx by private payors, employers, and health systems is increasing (75, 81, 99, 102).

## 4 Conclusion and future perspectives

DTx provide evidence-based interventions for disease prevention, treatment, or management. Using digital technologies, they enable access to care from nearly anywhere, at any time. However, there are considerations for different stakeholders and barriers that must still be overcome for DTx to receive wide implementation across clinical practice. Issues include lack of knowledge about DTx among clinicians and patients, coverage/reimbursement challenges, and insufficient infrastructure for adoption.

Although many DTx are available or under development, several questions critical to integration remain to be answered. Although there are no precedent court decisions on the new liability issues that may arise with DTx, malpractice liability is a potential issue (103). Errors in the design or malfunctioning software or hardware may have harmful consequences, particularly for the vulnerable populations with mental illnesses. While the manufacturer is responsible for the DTx functionality, HCPs are responsible for the selection and monitoring. It is unknown whether DTx create opportunities for harm since the long-term health risks are yet unclear.

As DTx are a relatively nascent treatment modality, there is still much to learn about which patients would benefit most and how to effectively deliver these treatments at scale. While early clinical research on DTx is encouraging, studies have varied widely in design and outcomes measured, necessitating for larger, longer, and more consistent research efforts to better evaluate their real-world effectiveness and considerations of how traditional research paradigms may or may not fit the characteristics of digital treatments. Through continued research and implementation, it should be possible to design better tailored comprehensive services that integrate digital tools in the future. Integration of DTx into EMR could increase utility and adoption.

While the adoption of DTx for mental health is expanding, global access remains uneven due to the absence of a harmonized international regulatory framework, with reimbursement and approval rules varying significantly by country. Outside of the US, Germany has emerged as a model, integrating digital health applications into its public health system for 73 million citizens, prompting similar initiatives in France, Belgium, and Austria (104, 105). Nevertheless, overall adoption remains low, and broader alignment of regulatory and reimbursement policies across jurisdictions will be essential to improving equitable access. In the UK, the Medicines and Healthcare products Regulatory Agency (MHRA) in partnership with the National Institute for Health and Care Excellence (NICE) have developed guidance for the regulation and evaluation of digital mental health technology to ensure only safe and effective products are made available (106). To date three digital health technologies have been recommended to help manage symptoms of psychosis and prevent relapse in adults and young people (AVATAR, SlowMo, and CareLoop) (107).

This manuscript has several limitations. As a narrative review and expert perspective, it does not represent a systematic review of the literature or a formal evaluation of the quality of available evidence. The recommendations presented are informed by the current evidence base, recent policy developments, and the authors’ clinical implementation experience and may not be generalizable to all healthcare settings or jurisdictions. In addition, the field of DTx is evolving rapidly, with ongoing changes in clinical evidence, regulatory oversight, reimbursement policies, and available technologies. Consequently, some recommendations may require refinement as new evidence and implementation experiences emerge.

Overall, DTx can provide valuable psychosocial intervention for people with severe mental illnesses. With expanding evidence of clinical effectiveness and safety as well as increased awareness, coverage/reimbursement will become more available. Care team members are well-positioned to advocate for patients to have access to DTx, empower patients to seek this treatment, and support them to achieve optimal outcomes from DTx. Advances in technology will facilitate further expansion of digital care, and while many existing DTx are based on psychosocial interventions, advancements may facilitate the development of novel interventions.

## Data Availability

The original contributions presented in the study are included in the article/Supplementary material, further inquiries can be directed to the corresponding authors.
