# Supplementary material for: Integrating digital therapeutics for patients with mental illness into clinical practice: perspectives for US healthcare professionals
Source: Front Public Health. 2026 Sep 2;14:1817617. doi: 10.3389/fpubh.2026.1817617 (PMC13579911; doi:10.3389/fpubh.2026.1817617)

Supplemental Table 1. FDA-reviewed DTx for mental health treatment

Product	Indication	Description	Authorization type
**reSET®<br>(15, 16, 102)**	SUD; adjunct to usual care in adults ≥18 years of age	Smartphone app including core modules focused on CBT and relapse-prevention skills, as an adjunct to outpatient treatment. Commercial adaptation of the Therapeutic Education System, an internet-based program including Contingency Management and Community Reinforcement Approach to CBT	De Novo authorization (2017)
**reSET-O®<br>(17-19, 103)**	OUD; adjunct to usual care in adults ≥18 years of age	Smartphone app including core modules focused on CBT and relapse-prevention skills, as an adjunct to outpatient treatment. Commercial adaptation of the Therapeutic Education System, an internet-based program including Contingency Management and Community Reinforcement Approach to CBT	510(k) clearance (2018)
**Freespira®<br>(20-22, 104)**	PTSD and panic disorder; adjunct to usual care in adults ≥18 years of age	Treatment for anxiety attacks and PTSD symptoms involving the Capnometry Guided Respiratory Intervention System, including a breathing sensor and tablet with app, for correction of dysfunctional breathing	510(k) clearance (2018)
**Somryst™<br>(28-31, 105)**	Chronic insomnia; adults ≥22 years of age	Mobile app delivering CBT for insomnia, including sleep restriction, stimulus control, cognitive restructuring, sleep hygiene, and relapse prevention	510(k) clearance (2020)
**EndeavorRx®<br>(24, 25, 106, 107)**	ADHD; adjunct to usual care in children 8–17 years of age	Video game accessed on mobile devices with sensory stimuli and motor challenges targeting areas of the brain that play a key role in attention function	De Novo authorization (2020)
**EndeavorOTC<br>(108, 109)**	ADHD; adjunct to usual care in adults ≥18 years of age	Video game accessed on mobile devices with sensory stimuli and motor challenges targeting areas of the brain that play a key role in attention function	510(k) clearance (2024)

**NightWare™ (32, 33, 110-112)**	Nightmare disorder or nightmares from PTSD; adults ≥22 years of age	Smartwatch-based application providing vibrotactile feedback based on an analysis of heart rate and motion during sleep to interrupt nightmares without waking the patient	De Novo authorization (2020)
**DaylightRx (23, 113)**	GAD; adjunct to usual care in adults ≥22 years of age	Smartphone app targeting anxiety symptoms with a personalized digital CBT program including modules based on applied relaxation, stimulus control, cognitive restructuring, and imaginal exposure principles	510(k) clearance (2024)
**Rejoyn™ (27, 89, 90, 114)**	MDD; adjunct to usual care in adults ≥22 years of age who are on antidepressant medication	Smartphone app targeting depressive symptoms. Cognitive-emotional and behavioral interventions including Emotional Faces Memory Task, lessons, and activities for therapeutic skills	510(k) clearance (2024)
**SleepioRx (115-118)**	Chronic insomnia/insomnia disorder; adjunct to usual care in adults ≥18 years of age	Mobile app delivering CBT for insomnia, including sleep restriction, stimulus control, cognitive therapy, relaxation techniques, and sleep hygiene and education	510(k) clearance (2024)
**MamaLift Plus™ (119, 120)**	Mild-to-moderate postpartum depression; adjunct to usual care in adults ≥22 years of age	Smartphone app delivering digital CBT, Behavioral Activation Therapy, Interpersonal Therapy, and Dialectical Behavior Therapy, as an adjunct to outpatient treatment	510(k) clearance (2024)

ADHD, attention-deficit/hyperactivity disorder; app, application; CBT, cognitive behavioral therapy; FDA, US Food and Drug Administration; GAD, generalized anxiety disorder; MDD, major depressive disorder; OUD, opioid use disorders; PTSD, post-traumatic stress disorder; SUD, substance use disorders.

Supplemental Figure 1. Patient-facing digital health product types (1, 2)

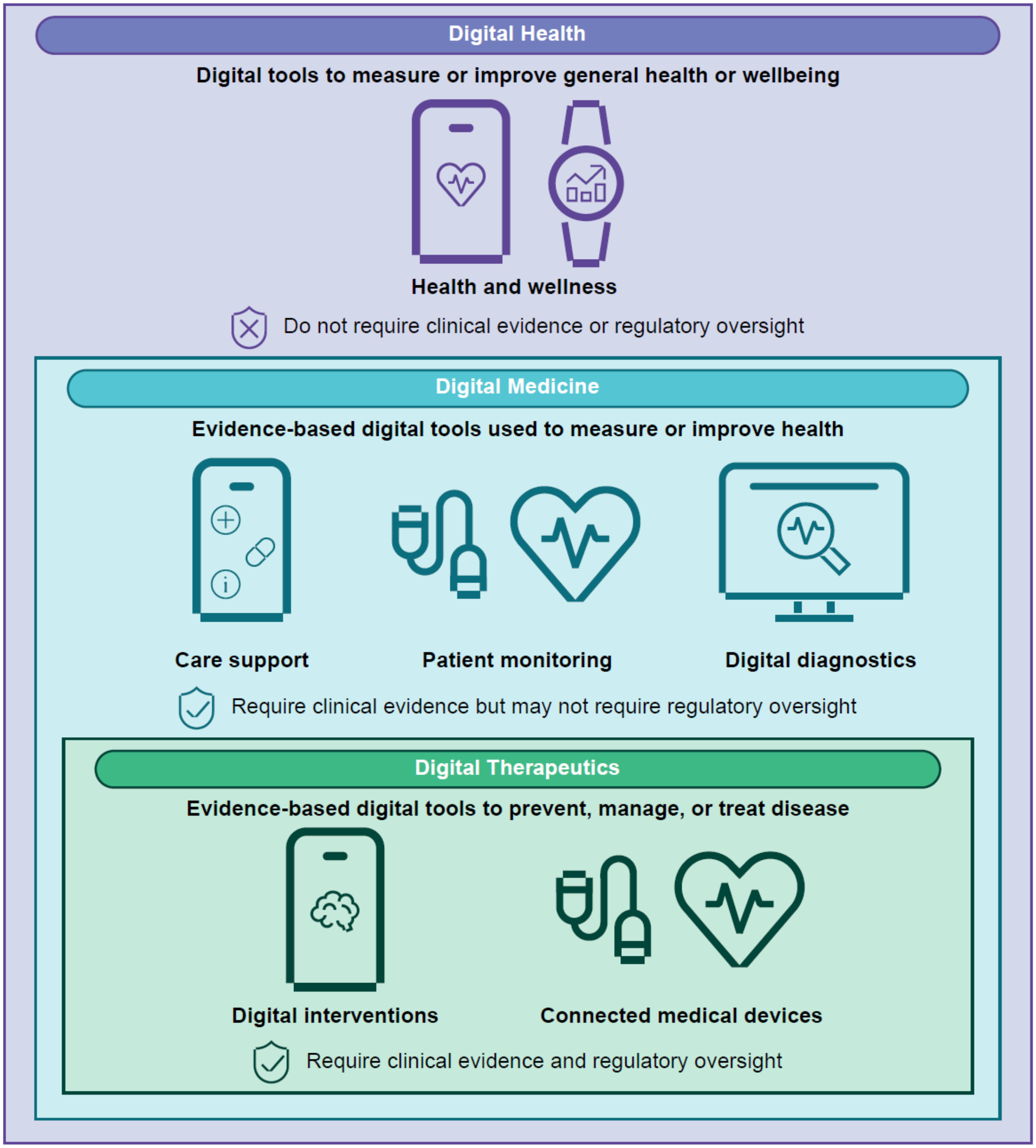

Supplement: Supplementary file 1 [file Data_Sheet_1.pdf]
